# Aflatoxin B1-DNA adducts modify the effects of post-operative adjuvant transarterial chemoembolization improving hepatocellular carcinoma prognosis

**DOI:** 10.37349/etat.2023.00167

**Published:** 2023-08-31

**Authors:** Liyan Huang, Qinqin Long, Qunying Su, Xiaoying Zhu, Xidai Long

**Affiliations:** The First Clinical Medical College of Lanzhou University, China; ^1^Clinicopathological Diagnosis & Research Centre, The Affiliated Hospital of Youjiang Medical University for Nationalities, Baise 533000, Guangxi Zhuang Autonomous Region, China; ^2^Departement of Pathology, Graduate School of Youjiang Medical University for Nationalities, Baise 533000, Guangxi Zhuang Autonomous Region, China

**Keywords:** Aflatoxin B1-DNA adduct, DNA damage, hepatocellular carcinoma, post-operative adjuvant transarterial chemoembolization, prognosis

## Abstract

**Aim:**

DNA damage involves in the carcinogenesis of some cancer and may act as a target for therapeutic intervention of cancers. However, it is unclear whether aflatoxin B1 (AFB1)-DNA adducts (ADAs), an important kind of DNA damage caused by AFB1, affect the efficiency of post-operative adjuvant transarterial chemoembolization (po-TACE) treatment improving hepatocellular carcinoma (HCC) survival.

**Methods:**

A hospital-based retrospective study, including 318 patients with Barcelona Clinic Liver Cancer (BCLC)-C stage HCC from high AFB1 exposure areas, to investigate the potential effects of ADAs in the tissues with HCC on po-TACE treatment. The amount of ADAs in the cancerous tissues was tested by competitive enzyme-linked immunosorbent assay (c-ELISA).

**Results:**

Among these patients with HCC, the average amount of ADAs was 3.00 µmol/mol ± 1.51 µmol/mol DNA in their tissues with cancer. For these patients, increasing amount of ADAs was significantly associated with poorer overall survival (OS) and tumor reoccurrence-free survival (RFS), with corresponding death risk (DR) of 3.69 (2.78–4.91) and tumor recurrence risk (TRR) of 2.95 (2.24–3.88). The po-TACE therapy can efficiently improve their prognosis [DR = 0.59 (0.46–0.76), TRR = 0.63 (0.49–0.82)]. Interestingly, this improving role was more noticeable among these patients with high ADAs [DR = 0.36 (0.24–0.53), TRR = 0.40 (0.28–0.59)], but not among those with low ADAs (*P* > 0.05).

**Conclusions:**

These results suggest that increasing ADAs in the cancerous tissues may be beneficial for po-TACE in ameliorating the survival of patients with HCC.

## Introduction

Hepatocellular carcinoma (HCC) is one of the most common malignant tumors with high incidence and death rate in the worldwide. Because HCC often displays its’ high early-stage blood metastasis and recurrence risk, patients with HCC usually feature a poor prognosis. With advancing in the scientific technology, some new therapeutic methods, including the transarterial chemoembolization (TACE), radiofrequency ablation, microwave ablation, percutaneous ethanol ablation, targeting therapy and radioembolization, have been developed and utilized in some HCC cases. Among these therapeutic methods, TACE treatment has been reported widely to be able to effectively improve the survival status of patients with HCC, especially these with advanced-stage tumor. In China, post-operative adjuvant TACE (po-TACE) treatment has been recommended as an important post-operative adjuvant treatment for HCC patients with high risk of tumor intrahepatic metastasis. However, increasing evidence has shown that po-TACE treatment displays different therapeutic effects on patients with different genetic profiles or biomarkers. Thus, it is vital to discern specific biomarkers or genetic profiles for improving the therapeutic effects of po-TACE treatment on HCC prognosis.

Aflatoxin B1 (AFB1) is an important I-type chemical carcinogen, which can induce HCC [1]. Growing evidence has shown that AFB1-caused DNA damage, including AFB1-DNA adducts (ADAs), single-strand breaks (SSBs), and double-strand breaks (DSBs), play a vital role in the carcinogenesis of AFB1-induced HCC. DNA damage induced by AFB1, especially ADAs, will change the structures of DNA sequence and lead to mutations of some genes such as TP53 and Ras [1–3]. On the one hand, this kind of accumulating gene alterations may disrupt corresponding encoding-protein structures and functions and affect the capacity of DNA damage response (DDR), which can result in harmful effects on human health, such as aging, senescence, abnormal apoptosis status, genomic instability, and ultimately severe cancer progression in normal cells [1, 4]. On the other hand, DNA damage-linked abnormal DDR capacity occurring in cancer cells may be regarded as potential targets for cancer therapy [5–11].

However, it is unclear whether ADAs in HCC modify the effects of po-TACE treatment improving HCC survival. A retrospective study was conducted to investigate the association between the amount of ADAs in the cancerous tissues with HCC and the therapeutic effects of po-TACE treatment on HCC.

## Materials and methods

### Patients

This study was a hospital-based retrospective study from Guangxi area (a high AFB1 exposure area). The inclusion criteria on cases include: (1) HCCs diagnosed by histopathological method; (2) tumor being in the Barcelona Clinic Liver Cancer (BCLC)-C stage; (3) cases having Child-Pugh A-stage liver function; (4) cases receiving partial liver resect or partial liver resect plus po-TACE as initial treatment according to Chinese Manage Criteria of Hepatocarcinoma (CMCH); (5) cases approving of this study and without dropping out; and (6) cases with available clinicopathological data, survival data, and cancerous tissue samples. The exclusion criteria on cases are the following: (1) cases with the history of radiotherapy or chemotherapy before receiving surgical resect therapy; (2) pregnant or breastfeeding women cases; and (3) cases with other tumors except HCC.

Based on the above-mentioned inclusion and exclusion criteria on cases, a total of 318 HCC patients (including 116 cases previously studied [12]), were recruited in the affiliated hospitals of Youjiang Medical University for Nationalities and Guangxi Medical University between May 2008 and May 2013. In this study, the response rate for cases with HCC was about 97.7%. Among these patients, 158 of them received po-TACE treatment as adjuvant therapy in the initial treatment procedure and were defined po-TACE group; others were defined as a control group (*n* = 160).

After written consent was obtained, all baseline clinicopathological features of all cases, including gender, age, ethnicity, smoking and drinking status, hepatitis B virus (HBV) and hepatitis C virus (HCV) infective status, liver cirrhosis, tumor grade and stage, and treatment information, were obtained as described previously [13]. At the same time, tissular samples with HCC of all patients were collected for analyzing the amount of ADAs and microvessel density (MVD). In this study, HBV and HCV infective status was evaluated using hepatitis surface antigen and anti-HCV antibody, respectively, whereas liver cirrhosis was estimated through pathological biopsy technique. Tumor grades were defined according to Edmondson and Steiner (ES) grading system.

For survival analyses, all patients with HCC underwent serial monitoring as described previously [14, 15]. In this study, the last following-up date was March 30, 2022. The duration of overall survival (OS) was defined as from the date of initial treatment to the date of death or last known date alive, whereas the tumor recurrence-free survival (RFS) was defined as from the date of initial treatment to the date of tumor recurrence or last known date alive.

### Po-TACE procedure

In this study, po-TACE was regarded as an adjuvant treatment procedure of the initial therapeutic procedure for eligible patients with HCC as described previously [12]. In brief, this procedure, containing an injection of mixture solution of cisplatin (7 mg/m^2^, BOLONG, Nanjing Pharmaceutical Factory Co., Ltd, China), doxorubicin (65 mg/m^2^, WANLE, Shenzhen Main Luck Pharmaceuticals Inc., China) and lipiodol, started 4 weeks after the removal therapy of tumor.

### ADAs assays

The amount of ADAs in the tissues with HCC was calculated by competitive enzyme-linked immunosorbent assay (c-ELISA) as described previously [16]. Briefly, DNA samples were first extracted from the cancerous tissues and dissolved in 1× PBS solution. Next, DNA samples were denatured and the ADAs in these samples were quantitated by c-ELISA method using 6A10 antibody (cat#NB600-443, Novus Biologicals, LLC). For analysis, the levels of ADAs were divided into two groups: low (≤ 3.00 µmol/mol DNA) and high (> 3.00 µmol/mol DNA), according to the average value of ADAs among patients with HCC.

### MVD evaluation

MVD in the tumor was elucidated using immunohistochemistry staining of CD34 (cat#2011101101, Gene Tech., Shanghai, China) as described previously [16]. To analyze the relationship between ADAs and MVD, MVD status was divided into two groups: low (≤ 50/×200 magnifications) and high (> 50/×200 magnifications), according to its mean of tumor vessels.

### Statistical analysis

All statistical analyses were finished by the Statistical Package for Social Science (SPSS) version 18 (SPSS Institute, Chicago, IL). The difference between groups was tested by Pearson chi-squared test (*χ*^2^) or *t* test, whereas the link between ADAs and MVD in the cancerous tissues were analyzed by Spearman’s correlation method. The effects of every variable on HCC survival were elucidated using Kaplan-Meier survival model (with Log-rank test) and Cox regression survival model (including univariable and multivariable models). Median survival time, death risk (DR) and tumor recurrence risk (TRR) were used for evaluating the prognostic potential of variables. In this study, a value of *P* < 0.05 was considered statistically significant.

## Results

### The clinicopathology information of HCC patients

A total of 318 patients with BCLC-C stage HCC were included in the final analyses. The Table 1 summarized the distribution of clinicopathological characters among po-TACE treatment group and controls and results showed that these baseline clinicopathological features were matched between two groups (*P* > 0.05).

**Table 1:** The clinicopathological features of HCC cases with or without po-TACE treatment

**Variable**	**Controls (*n* = 160)**		**Po-TACEs (*n* = 158)**		***χ* ^2^**	***P***
	***n***	**%**	***n***	**%**		
Age (years old)^a^						
48	91	56.9	88	55.7	0.045	0.832
> 48	69	43.1	70	44.3		
Gender						
Male	113	70.6	102	64.6	1.337	0.248
Female	47	29.4	56	35.4		
Race						
Han	93	58.1	79	50.0	2.113	0.146
Zhuang	67	41.9	79	50.0		
HBV status						
Negative	47	29.4	54	34.2	0.846	0.358
Positive	113	70.6	104	65.8		
HCV status						
Negative	146	91.3	137	86.7	1.674	0.196
Positive	14	8.8	21	13.3		
Smoking status						
No	121	75.6	121	76.6	0.040	0.841
Yes	39	24.4	37	23.4		
Drinking status						
No	113	70.6	122	77.2	1.790	0.181
Yes	47	29.4	36	22.8		
AFP (ng/L)						
20	57	35.6	59	37.3	0.101	0.750
> 20	103	64.4	99	62.7		
Liver cirrhosis						
No	37	23.1	40	25.3	0.208	0.648
Yes	123	76.9	118	74.7		
ES grade^b^						
Low	80	50.0	88	55.7	1.036	0.309
High	80	50.0	70	44.3		
MVD						
No	55	34.4	56	35.4	0.040	0.842
Yes	105	65.6	102	64.6		

^a^ Age is grouped according to the average age of patients with HCC (47.94 years old 9.98 years old); ^b^ ES grade is divided into two groups: low grade (ES-I and -II grade) and high grade (ES-III and -IV grade). AFP: α-fetoprotein

### Po-TACE therapy improving the outcomes of HCC

**Figure 1:**
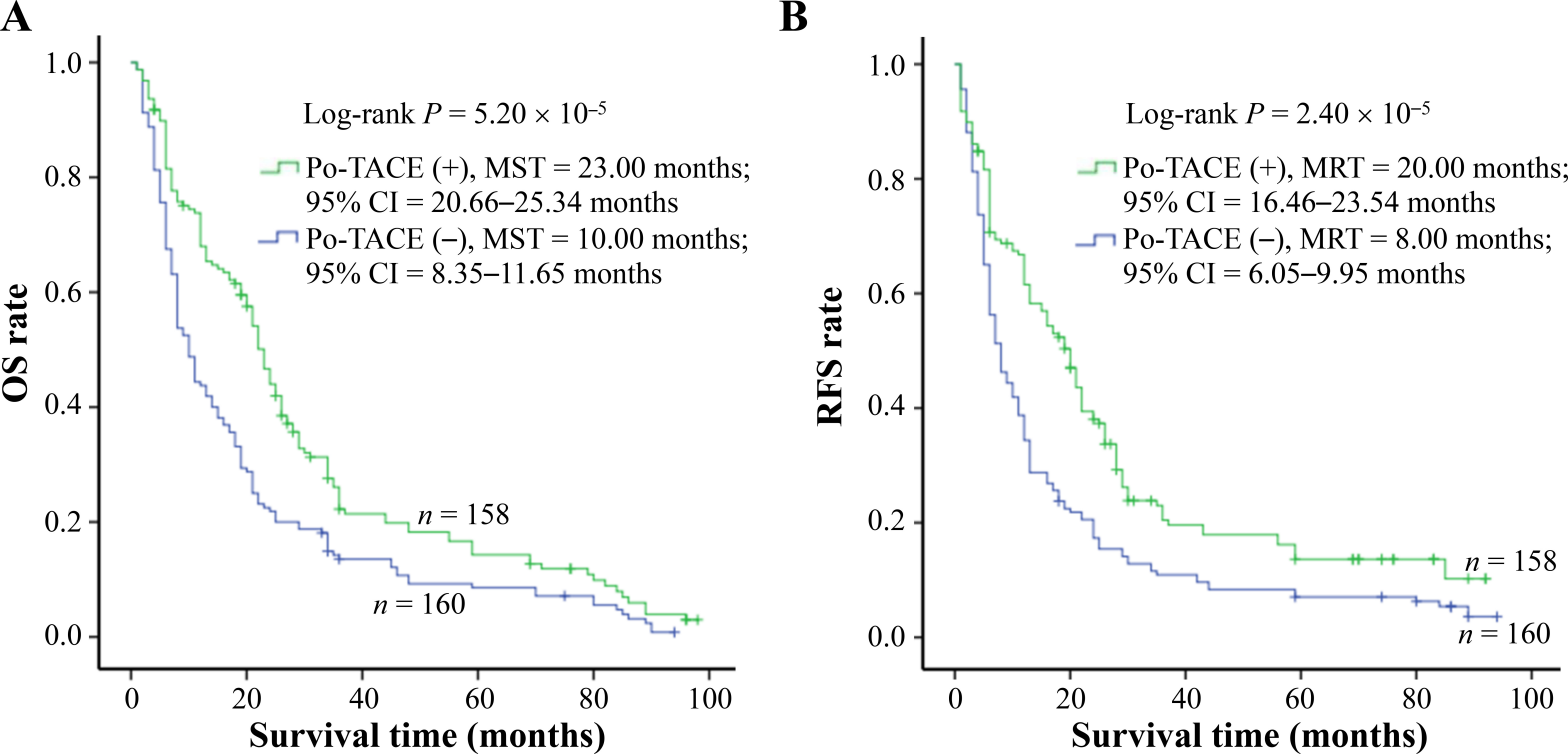
Po-TACE treatment significantly correlating with HCC survival. Po-TACE treatment is associated with (A) OS and (B) tumor RFS of HCC. Cumulative hazard function was plotted by Kaplan-Meier’s methodology, and *P* value was calculated with two-sided Log-rank tests. (+): patients receiving po-TACE treatment; (−): patients without receiving po-TACE treatment; CI: confidence interval

Results from Kaplan-Meier survival models displayed that these HCC patients receiving po-TACE treatment featured a better OS compared to those not doing po-TACE treatment (Figure 1A) and RFS (Figure 1B). Median survival time (MST) was 23.00 months and 10.00 months for po-TACE and control group, respectively, whereas median tumor recurrence-free time (MRT) was 20.00 months and 8.00 months, respectively. Univariate Cox regression survival model analyses showed that compared with the control group, po-TACE treatment significantly decreased DR [0.63 (0.50–0.80)] and TRR [0.61 (0.48–0.78)] of patients with HCC (Table 2). Results from multivariate Cox regression survival model analyses further proved that po-TACE therapy significantly improved the survival of HCCs (Table 3).

**Table 2:** Univariate analyses of potential viables for survival of patients with HCC

**Variables**	**OS**		**RFS**	
	**DR (95% CI)**	***P*_trend_**	**TRR (95% CI)**	***P*_trend_**
Age (> 48 years old *vs. * 48 years old)^a^	0.89 (0.70–1.12)	0.31	0.88 (0.69–1.12)	0.29
Gender (female *vs.* male)	0.91 (0.71–1.17)	0.47	0.90 (0.70–1.15)	0.39
Ethnicity (Zhuang *vs.* Han)	0.81 (0.64–1.02)	0.07	0.77 (0.61–1.08)	0.08
Smoking (yes *vs.* no)	0.76 (0.58–0.99)	0.05	0.76 (0.58–1.01)	0.06
Drinking (yes *vs.* no)	0.86 (0.66–1.12)	0.26	0.82 (0.63–1.08)	0.16
HBV status (positive *vs.* negative)	1.04 (0.81–1.33)	0.79	1.20 (0.93–1.55)	0.16
HCV status (positive *vs.* negative)	1.03 (0.72–1.48)	0.86	1.03 (0.71–1.50)	0.87
AFP (> 20 ng/mL *vs. * 20 ng/mL)	1.11 (0.88–1.41)	0.38	1.21 (0.95–1.55)	0.13
Liver cirrhosis (yes *vs.* no)	1.20 (0.92–1.58)	0.19	1.13 (0.85–1.49)	0.40
ES grade (high *vs.* low)	1.48 (1.17–1.87)	8.99 10^–4^	1.57 (1.24–1.99)	2.11 × 10^–4^
MVD (positive *vs.* negative)	1.39 (1.09–1.78)	7.30 10^–5^	1.22 (0.96–1.57)	0.11
Po-TACE treatment (yes *vs.* no)	0.63 (0.50–0.80)	9.80 10^–5^	0.61 (0.48–0.78)	5.10 × 10^–5^
ADAs (high *vs.* low)^b^	3.89 (2.95–5.12)	4.66 10^–22^	3.08 (2.36–4.02)	1.10 × 10^–16^

^a^ Age is grouped according to the average age of patients with HCC (47.94 years old 9.98 years old); ^b^ ADAs are grouped according to the average ADA of patients with HCC (3.00 µmol/mol 1.51 µmol/mol DNA). *P*_trend_: *P* value is calculated by trend test in the models

**Table 3:** Multivariate analyses of potential viables for survival of patients with HCC

**Variables**	**OS**		**RFS**	
	**DR (95% CI)**	***P*_trend_**	**TRR (95% CI)**	***P*_trend_**
Age (> 48 years old *vs. * 48 years old)^a^	0.94 (0.72–1.18)	034	0.95 (0.70–1.28)	0.25
Gender (female *vs.* male)	1.00 (0.77–1.29)	0.99	0.96 (0.74–1.25)	0.76
Ethnicity (Zhuang *vs.* Han)	0.89 (0.70–1.13)	0.34	0.79 (0.61–1.01)	0.06
Smoking (yes *vs.* no)	0.73 (0.52–1.02)	0.07	0.83 (0.59–1.17)	0.28
Drinking (yes *vs.* no)	0.95 (0.69–1.32)	0.78	0.82 (0.58–1.15)	0.25
HBV status (positive *vs.* negative)	0.98 (0.75–1.29)	0.90	1.16 (0.88–1.53)	0.29
HCV status (positive *vs.* negative)	1.01 (0.69–1.49)	0.963	1.00 (0.68–1.49)	0.99
AFP (> 20 ng/mL *vs. * 20 ng/mL)	1.22 (0.95–1.56)	0.13	1.31 (1.01–1.69)	0.04
Liver cirrhosis (yes *vs.* no)	1.02 (0.76–1.36)	0.91	0.97 (0.72–1.29)	0.81
ES grade (high *vs.* low)	1.31 (1.03–1.68)	0.03	1.39 (1.09–1.79)	9.14 × 10^–3^
MVD (positive *vs.* negative)	1.24 (0.95–1.62)	0.12	1.09 (0.83–1.42)	0.53
Po-TACE treatment (yes *vs.* no)	0.59 (0.46–0.76)	5.10 10^–5^	0.63 (0.49–0.82)	4.36 × 10^–4^
ADAs (high *vs.* low)^b^	3.69 (2.78–4.91)	2.02 10^–19^	2.95 (2.24–3.88)	1.52 × 10^–14^

^a^ Age is grouped according to the average age of patients with HCC (47.94 years old 9.98 years old); ^b^ ADAs are grouped according to the average ADA of patients with HCC (3.00 µmol/mol 1.51 µmol/mol DNA). *P*_trend_: *P* value is calculated by trend test in the models

### ADAs significantly affecting HCC prognosis

**Figure 2:**
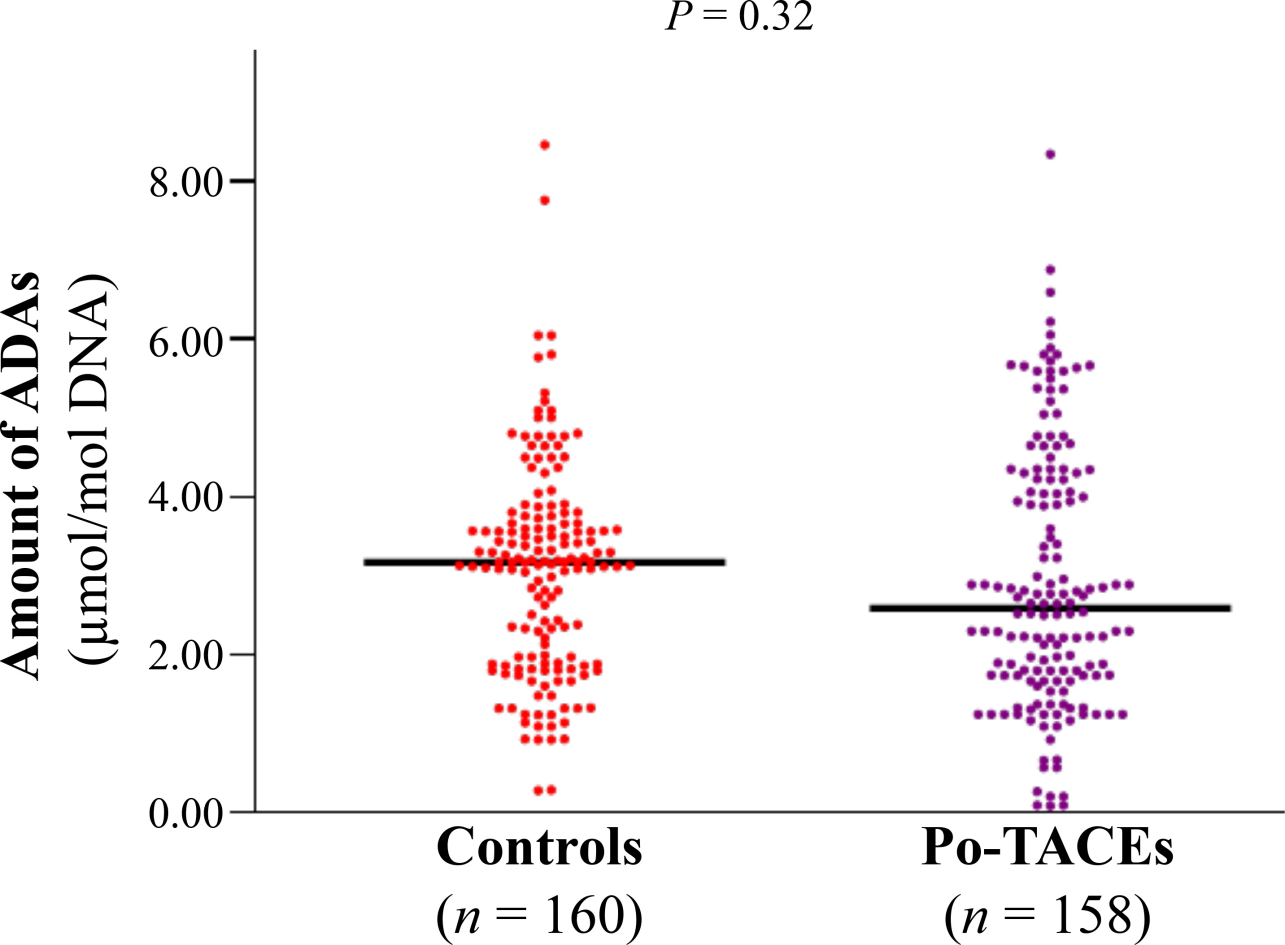
The distribution of ADAs between po-TACE group and control group. The amount of ADAs in the cancerous tissues with HCC is calculated by c-ELISA and the difference for the distribution of ADAs between two groups is tested by *t* test. Black lines represent the mean of ADAs in each group

**Figure 3:**
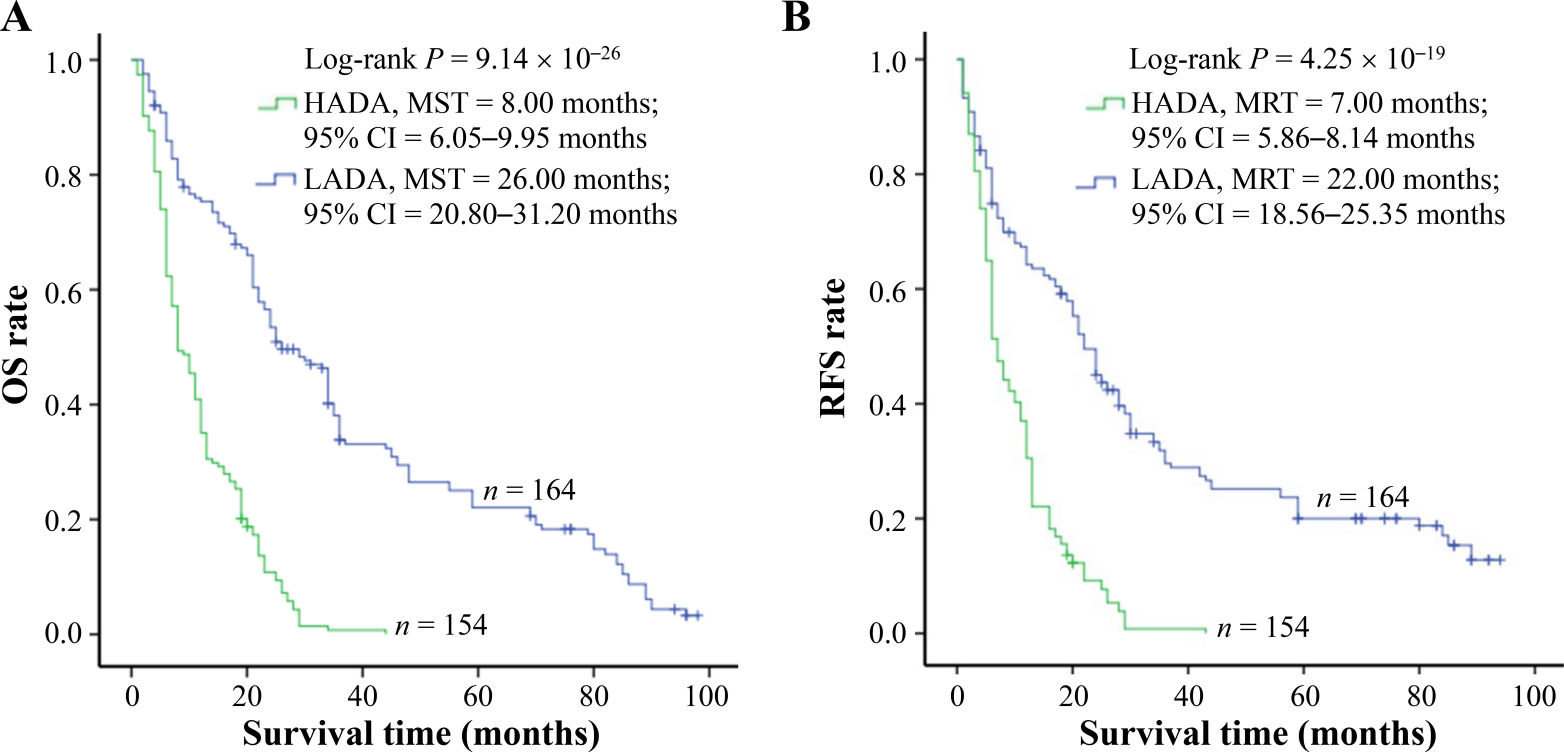
ADAs significantly correlating with HCC survival. The amount of ADAs in the cancerous tissues with HCC is calculated by c-ELISA. To analyze, the levels of ADAs are divided into two groups: LADA and HADA. ADAs are associated with (A) OS and (B) tumor RFS of HCC. Cumulative hazard function was plotted by Kaplan-Meier’s methodology, and *P* value was calculated with two-sided Log-rank tests

The amount of ADAs was elucidated by enzyme-linked immunosorbent assay (ELISA) assay and a similar amount of ADAs was tested in cancerous tissue samples from control and po-TACE group (3.09 µmol/mol ± 1.34 µmol/mol *vs.* 2.92 µmol/mol ± 1.66 µmol/mol DNA) (Figure 2). To analyze the effects of ADAs on HCC survival, ADAs were divided into two groups: high level of ADAs (HADA) and low level of ADAs (LADA) according to the average amount of ADAs in tissue samples from all patients (3.00 µmol/mol 1.51 µmol/mol DNA). Results from Kaplan-Meier survival models displayed that these HCC patients with HADA in their cancerous tissues featured a poorer OS compared to those with LADA (Figure 3A) and RFS (Figure 3B). MST and MRT were 8.00 months *vs.* 26.00 months and 7.00 months *vs.* 22.00 months, respectively. Univariate Cox regression survival model analyses showed that compared with LADA, HADA significantly increased DR [3.89 (2.95–5.12)] and TRR [3.08 (2.36–4.02)] of patients with HCC (Table 2). Results from multivariate Cox regression survival model analyses further proved that ADA was a potential prognostic marker for HCC (Table 3).

### ADAs differentially modified po-TACE therapy in improving HCC prognosis

**Figure 4:**
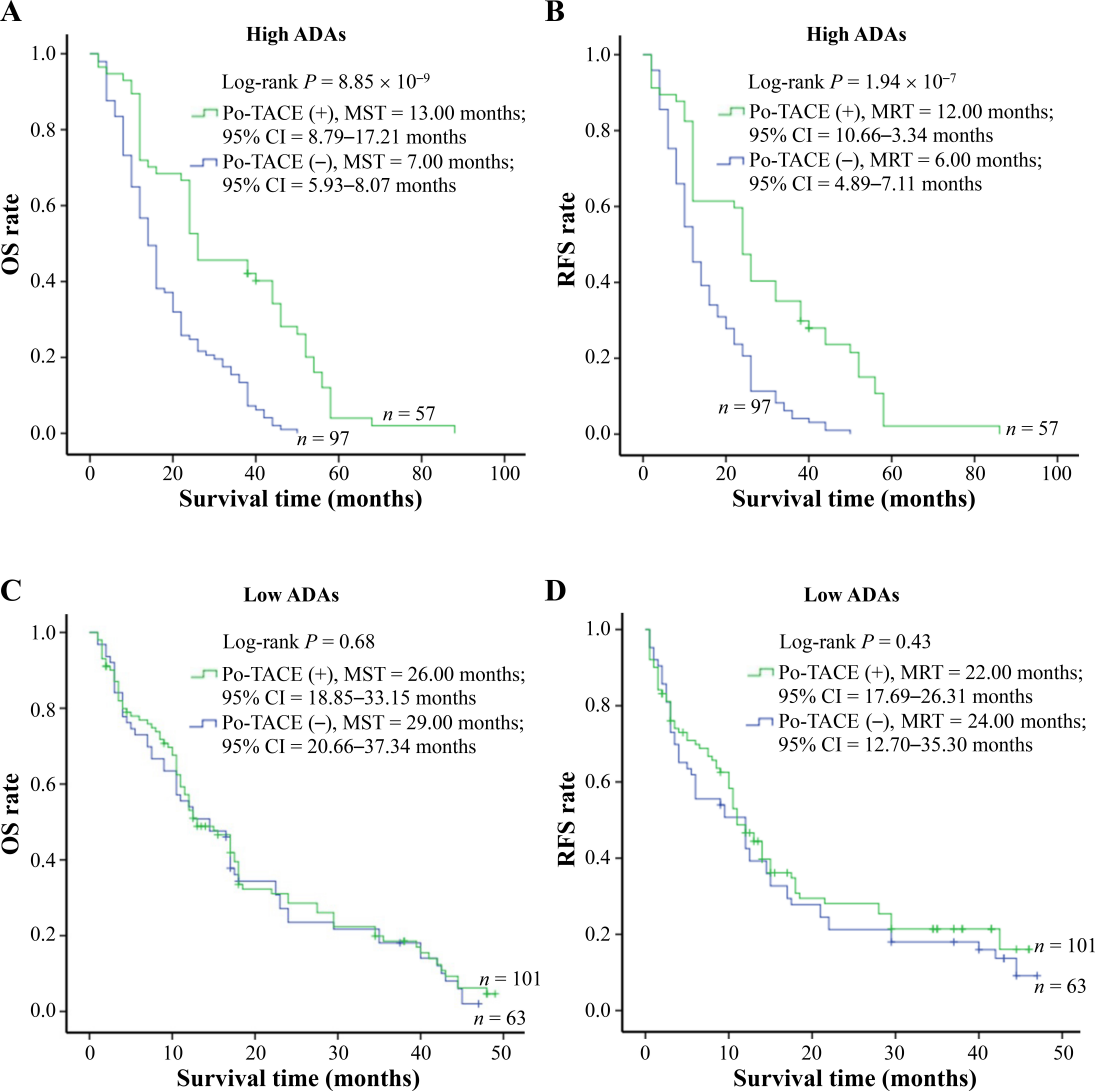
Survival analysis of po-TACE treatment in strata of ADAs. Among HCC cases with HADA, po-TACE treatment improved their (A) OS and (B) TRR. However, this treatment did not improve the (C) OS and (D) TRFS of those with LADA. Cumulative hazard function was plotted by Kaplan-Meier’s methodology, and *P* value was calculated with two-sided Log-rank tests. (+): patients receiving po-TACE treatment; (−): patients without receiving po-TACE treatment

In view of some studies showing that DNA damage may act as a potential target for therapeutic intervention [5, 17, 18], the present study explored whether ADAs modified the therapeutic effects of po-TACE treatment on HCC through a stratified analysis based on the different levels of ADAs in the tissues with HCC (Table 4). The results showed that po-TACE therapy can significantly improve the outcome of these HCC patients with HADA in their cancerous tissues (MST, from 7 months to 13 months; MRT, from 6 months to 12 months) (Figure 4A and 4B). Results from multivariable Cox regression survival model analyses further proved that these patients feature a decreasing DR [0.36 (0.24–0.53)] and TRR [0.40 (0.28–0.59)] if they received po-TACE treatment (Table 4). However, this kind of improvement in the HCC prognosis was not observed among those with LADA (*P* > 0.05) (Figure 4C and 4D, Table 4). Taken together, these results implied that ADAs can differently modify the efficiency of po-TACE therapy in improving HCC prognosis.

**Table 4:** The effects of po-TACE treatment on HCC prognosis stratified by ADAs

**ADA**	**Po-TACE treatment**	**OS**		**RFS**	
		**DR (95% CI)^a^**	***P*_trend_**	**TRR (95% CI)^a^**	***P*_trend_**
Low	Yes *vs.* no	0.84 (0.58–1.20)	0.33	0.84 (0.58–1.23)	0.37
High	Yes *vs.* no	0.36 (0.24–0.53)	2.67 × 10^–7^	0.40 (0.28–0.59)	2.00 × 10^–6^

^a^ Adjusted by age, sex, race, HBV and HCV status, smoking and drinking status, AFP, liver cirrhosis, and tumor grade. *P*_trend_: *P* value is calculated by trend test in the models

### The amounts of ADAs positively correlated with MVD in the tumor tissues

**Figure 5:**
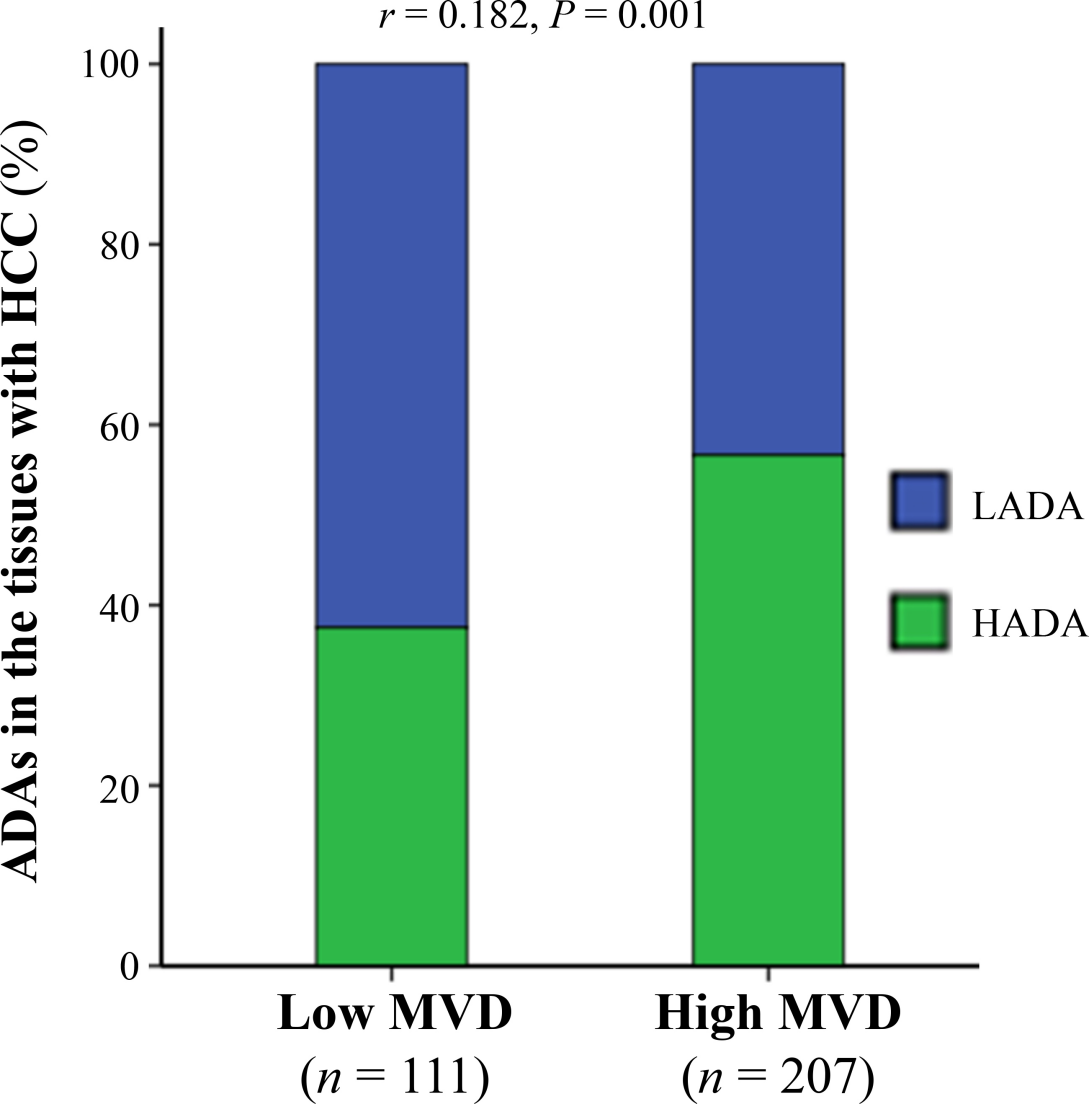
The association between ADAs in the cancerous tissues with HCC and tumor MVD. The data were analyzed using Spearman’s correlation analysis

Because of MVD in the tumor tissues positively correlating with therapeutic effects of po-TACE on HCC [19], this study investigated the association between ADAs and MVD (Figure 5). Results exhibited that the number of ADAs in the tissues with HCC was positively related to the amount of MVD in the tumor tissues (*r* = 0.182, *P* < 0.01).

## Discussion

To the best of authors’ knowledge, there is not a shred of evidence whether the DNA damage by AFB1 can act as a therapeutic target for HCC. On the basis of a retrospective study, this study investigated the potential association between ADA, an important DNA damage type induced by AFB, and the therapeutic efficiency of po-TACE treatment in improving HCC survival. It was found that increasing the amount of ADAs in the cancerous tissues with HCC had a significant correlation with the therapeutic efficiency of po-TACE on HCC. This study represents the first report indicating the possibility of ADA as a therapeutic target for po-TACE.

TACE treatment is an important therapeutic procedure for HCC [20]. Among known TACE techniques, conventional TACE (cTACE) and TACE with drug-eluting beads (DEB-TACE) are widely used worldwide. The cTACE, which commonly uses a mixture of lipoid and anticancer drugs (e.g., epirubicin, doxorubicin, or cisplatin), was developed and established in the late last century. In the procedure of cTACE treatment, emulsion mixtures are first injected into the tumor-feeding arteries; next, gelatin sponge or particles are administered. DEB-TACE technique has been developed, mainly because of enhancing the delivery of anti-cancer drugs, providing a better embolizing effect, and minimizing drug-related adverse events [20–22]. To date, drug-eluting beads (DEB) are designed as a kind of multifunction embolization microspheres. Furthermore, they are visible by imaging and have the capacity for loading different anticancer [20]. Evidence from *in vivo* and *in vitro* experiments and clinical trials has shown that TACE treatment can effectively improve the outcomes of patients with HCC [23, 24]. Today, TACE treatment has been recommended as the first treatment option for patients with intermediate-stage HCC (BCLC-B stage HCC) [22]. Additionally, this treatment is also widely used as a post-operative adjuvant treatment (namely po-TACE) for patients with advanced-stage HCC (BCLC-C stage HCC) [25, 26]. The present study also found that po-TACE treatment can significantly improve the survival of HCC patients after tumor resected. Compared with controls, these patients receiving po-TACE treatment featured a longer MST (10.00 months *vs.* 23.00 months) and MRT (8.00 months *vs.* 20.00 months) and a smaller DR (0.59, 95% CI = 0.46–0.76) and TRR (0.63, 95% CI = 0.49–0.82).

In the past decade, increasing evidence from clinical practice has shown that the therapeutic efficiency of po-TACE treatment can be modified by individuals’ genetic profiles and some potential biomarkers are identified for predicting the therapeutic efficiency of this treatment. This study investigated the predictive values of a DNA damage biomarker ADA for the effects of po-TACE treatment improving HCC survival through a hospital-based retrospective study and found ADAs in tumor samples with HCC can significantly modify the sensibility of HCC cases on po-TACE treatment. These patients with HADA will obtain a better survival under the conditions of po-TACE therapy than those with LADA. This may be because of the several reasons. First, increasing amount of ADAs in tumor tissues usually indicates the low capacity of detoxicating enzymes [12, 27, 28]. Evidence from molecular epidemiological studies has proved low detoxification capacity resulting from abnormal expression of detoxicating enzyme genes were positively correlated with the levels of ADAs in the peripheral blood or cancerous tissues with HCC [27, 28]. Furthermore, the downregulation of detoxicating enzyme cytochrome P450 (CYP450) 2W1 by microRNA-4651 significantly changed the efficiency of po-TACE treatment in improving HCC surviving [12]. Second, abnormal DNA repair capacity and the following DNA damage accumulation in cancer cells alters the response of cancer cells on anti-cancer drugs [5, 17, 29]. Molecular epidemiological studies conducted in high AFB1 exposure areas show that the genetic alterations in DNA repair genes (including XPC, XRCC4, XRCC1, and XPD) are not only associated with low DNA damage repair capacity and result in increasing amounts of ADAs, but also increase HCC risk and short survival of patients with HCC [15, 30–33]. For example, genetic alteration at the coding region of DNA repair gene XRCC4 increased the amount of ADAs in the HCC cells and change the sensibility of cancer cells on anticancer drug doxorubicin [34, 35]. Third, ADAs maybe affect the process of tumor angiogenesis. Evidence from clinicopathological studies has displayed that there is an increasing heterogenic expression profile of chromobox 4 (CBX4) in tumor tissular samples with HADA and this alteration statistically modifies the therapeutic efficiency of po-TACE treatment on HCC [19]. This may be result from the upregulation of CBX4 expression accelerating tumor angiogenesis and next inducing tumor proliferation and decreasing cancerous cells’ sensibility on anticancer drugs [14, 19, 36]. The present study furthermore proved that the levels of ADAs in tumor tissues were linked with tumor MVD. Similar findings are shown in genetic variations [12, 37]. Finally, ADAs can change the cancer phenotypes, such as proliferation, apoptosis, cell cycle, autophagy, and oxidative endoplasmic reticulum stress, via activating oncogenes and inducing mutations of tumor suppressor genes [2–4]. These phenotypes maybe promote tumor progression and modify the therapeutic efficiency of po-TACE treatment in improving HCC survival [38].

In conclusion, this study describes ADAs in tissues with HCC and its link with the therapeutic value of po-TACE treatment and displays that ADAs can act as a valuable biomarker for forecasting the therapeutic significance of po-TACE treatment. These findings indicate that analyzing the amount of ADAs in the cancerous tissues with HCC may help us form a management strategy to improve the survival of patients with HCC. However, the present study was based on a relatively small sample, which may limit the potential of ADAs predicting the therapeutic efficiency of po-TACE on HCC. Although the present study explored the relationship between ADAs and tumor MVD, the corresponding mechanism analyses were not finished. Thus, forthcoming studies in combination with a larger size sample and mechanism analyses will be required to fully evaluate the utility of ADAs in selecting patients for po-TACE therapy.

## Abbreviations

ADAs: aflatoxin B1-DNA adducts
AFB1: aflatoxin B1
BCLC: Barcelona Clinic Liver Cancer
CI: confidence interval
cTACE: conventional transarterial chemoembolization
DR: death risk
ES: Edmondson and Steiner
HADA: high level of aflatoxin B1-DNA adducts
HBV: hepatitis B virus
HCC: hepatocellular carcinoma
HCV: hepatitis C virus
LADA: low level of aflatoxin B1-DNA adducts
MRT: median tumor reoccurrence-free time
MST: median survival time
MVD: microvessel density
OS: overall survival
Po-TACE: post-operative adjuvant transarterial chemoembolization
RFS: recurrence-free survival
TACE: transarterial chemoembolization
TRR: tumor recurrence risk

## Declarations

### Copyright

© The Author(s) 2023.
